# *Candida albicans *virulence and drug-resistance requires the *O*-acyltransferase Gup1p

**DOI:** 10.1186/1471-2180-10-238

**Published:** 2010-09-15

**Authors:** Célia Ferreira, Sónia Silva, Fábio Faria-Oliveira, Eva Pinho, Mariana Henriques, Cândida Lucas

**Affiliations:** 1 CBMA (Centre of Molecular and Environmental Biology), Department of Biology, Portugal; 2 IBB (Institute for Biotechnology and Bioengineering), Department of Biological Engineering, University of Minho, Campus de Gualtar, 4710-057 Braga, Portugal

## Abstract

**Background:**

*GUP1 *gene was primarily identified in *Saccharomyces cerevisiae *being connected with glycerol uptake defects in association with osmotic stress response. Soon after, Gup1p was implicated in a complex and extensive series of phenotypes involving major cellular processes. These include membrane and wall maintenance, lipid composition, bud-site selection, cytoskeleton orientation, vacuole morphology, secretory/endocytic pathway, GPI anchors remodelling, and lipid-ordered domains assembly, which is compatible with their inclusion in the Membrane Bound O-acyl transferases (MBOAT) family. In mammals, it has been described as a negative regulator of the Sonic hedgehog pathway involved in morphogenesis, differentiation, proliferation, among other processes.

**Results:**

We show that *Candida albicans *Gup1p strongly interferes with the capacity of cells to develop hyphae, to adhere, to invade, and to form a biofilm, all of which are significant virulence factors. Furthermore, the mutant colonies exhibited an aberrant morphology/differentiation pattern. Identically to *S. cerevisiae*, Ca*gup1Δ *null mutant was more resistant to antifungals like fluconazole, ketoconazole, and clotrimazole, and displayed an abnormal even sterol distribution at the plasma membrane.

**Conclusions:**

This work is the first study in the opportunistic yeast *Candida albicans*, showing a role for the *GUP1 *gene in virulence as well as in the mechanisms underlying antifungal resistance. Moreover, its impact is even more significant since these results, taken together with all the knowledge about *GUP1 *gene (from *S. cerevisiae *and mammals) give consistence to the possibility that Gup1p may be part of a yeast morphogenic pathway parallel to the mammalian Hedgehog.

## Background

*Candida albicans *is a commensal of human microflora, residing at the oral cavity, the gastrointestinal tract, the vaginal and the urinary environments, that acts as an opportunistic pathogen [reviewed by 1]. *C. albicans *commonly causes infections such as denture stomatitis, thrush, and urinary tract-infections, but can also provoke more severe systemic infections. These are frequently life-threatening, in particular in immuno-compromised individuals, whose numbers are constantly increasing due to organ transplant, chemotherapy, or, more importantly, to the prevalence of AIDS and Hepatitis C [reviewed by [1]].

Given the limited number of suitable and effective antifungal drugs, together with increasing drug resistance of the pathogens, it is important that research community addresses, and ultimately discloses, the following yet unsolved questions: a) how the transformation from commensal to pathogen takes place, b) how it can be prevented, c) which are the mechanisms underlying antifungal drugs resistance. All of these culminate in the need to search for new and better agents that target fundamental biological processes and/or pathogenic determinants.

*C. albicans*, as most pathogens, has developed an effective battery of virulence factors and specific strategies to assist the ability to colonize host tissues, cause disease, and overcome host defences [reviewed by [2]]. An outstanding attribute of *C. albicans *biology is its capacity to grow in a diversity of morphological forms, ranging from unicellular budding yeast (blastospores), pseudohyphae, to true hyphae with parallel-sided walls [3-5]. The yeast-hyphae transition contributes to tissue invasion and to the escape from phagocyte cells after host internalization [6], and is therefore considered an important virulence factor [4,5,8-11]. Additionally, several other factors have been described in association with virulence, including the production of proteins that mediate adherence, the colonization and invasion of host tissues, the maintenance of cell wall integrity, phenotypic switching, and the avoidance of the host immune response [12-18]. Many of these virulence factors are glycosylphosphatidylinositol (GPI) - anchored proteins, which comprise 88% of all covalently linked cell wall proteins in *C. albicans *[14], many of which associated with the lipid-ordered domains. In spite of all these knowledge, we are still far from fully understanding the precise mechanism(s) driven by *Candida *switch from commensal to pathogen status.

*Saccharomyces cerevisiae GUP1 *(Sc*GUP1*) is a Membrane Bound *O*-acyltransferase (MBOAT) recently proposed to act in the metabolism of lipids, with critical consequences on the lipid-ordered domains stability and assembly [19]. These domains are formed by tight associations of ergosterol and sphingolipids, and aggregate specific proteins, GPI-anchored and non-GPI [19-21]. In accordance, Sc*GUP1 *has been implicated in the proper GPI-anchors remodelling [22]. Among various classes of lipids in *C. albicans*, membrane ergosterol is an important constituent, which is also the target of common antifungals like polyenes and azoles [23-25]. Therefore, the action of antifungals is affected by changes in the membrane lipid composition, as well as its order (fluidity) and asymmetry in general, and by ergosterol content/distribution in particular [19,23,24,26-28]. Our group has shown [19], that the Sc*gup1Δ *mutant displays a moderate sensitivity to sphingolipids biosynthesis inhibitors (SBIs), but a higher resistance to ergosterol biosynthesis inhibitors (EBIs), including azoles. Additionally, the same work shows that the Sc*gup1Δ *mutant presents an abnormal sterol distribution in the plasma membrane, as well as internal membranes. In fact, *GUP1 *in *S. cerevisiae *has revealed to have a vast pleiotropic nature [19,22,29-32]. In mammals it was described as a negative regulator of the N-terminal palmitoylation of Sonic hedgehog pathway [33], which controls morphogenesis, differentiation and patterning during embryogenesis, including proliferation and cell fate.

In order to explore the involvement of *CaGUP1 *in drug susceptibility, we tested the growth of Ca*gup1Δ *null mutant in the presence of these compounds. Although, in *C. albicans*, as in *S. cerevisiae*, it is not possible to identify the precise Gup1p acyltransferase dependent reaction/s, we show that the deletion of *GUP1 *in *C. albicans *changes ergosterol plasma membrane constitution/distribution, presenting an increased resistance to azoles. More importantly, CaGup1p strongly interferes with the capacity of cells to develop hyphae, to adhere, to invade, and to form biofilms, all of which are significant virulence factors. To our knowledge, this work is the first study with *GUP1 *gene in *Candida albicans*, and it clearly shows a role for Ca*GUP1 *gene in virulence.

## Results

### *CaGUP1 *deletion provokes resistance to antifungals

**Figure 1:**
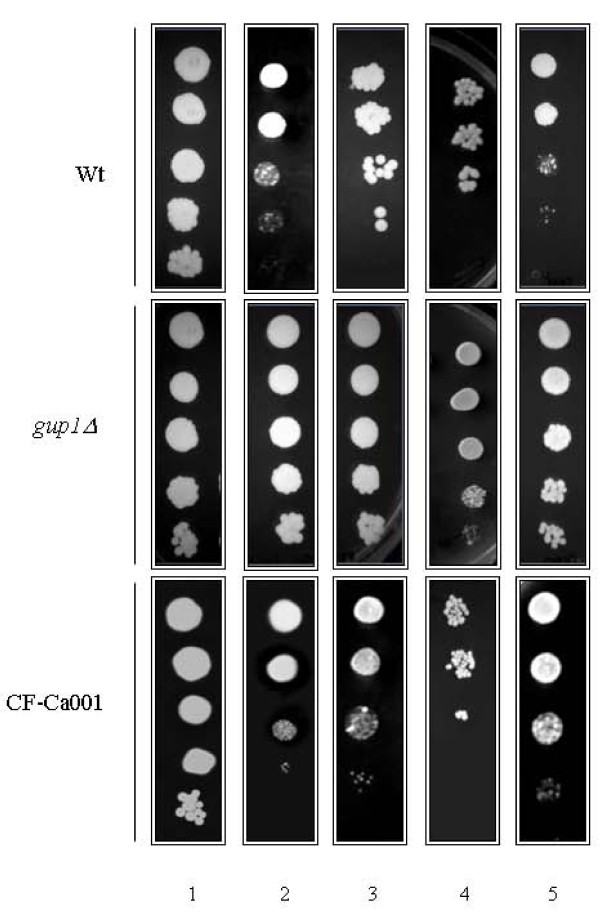
***Cagup1Δ *null mutant strain displays an altered sensitivity to specific ergosterol biosynthesis inhibitors**. Isogenic wt, Ca*gup1Δ *null mutant and CF-Ca001 strain were grown to mid-exponential phase in YPD medium. Ten-fold serial dilutions were spotted onto (1) YPD plates (control) and plates supplemented with (2) clotrimazole 68.8 μg/ml, (3) ketoconazole 106.3 μg/ml, (4) fluconazole 30.6 μg/ml and (5) fenpropimorph 60 μg/ml. All plates were incubated at 30°C and photographed after 3-5 days. The *gup1Δ *panel photos are representative of the results obtained with the several clones (3-5) of Ca*gup1Δ *null mutant strain tested.

The *S. cerevisiae O*-acyltransferase Gup1p acts on lipids metabolism affecting the plasma membrane sphingolipids-sterol ordered domains assembly/integrity, and influencing the susceptibility to antifungal drugs [19]. An association between altered lipid-ordered domains and antifungal resistance has been described before [23,24,34,35]. Therefore, we examined the growth behaviour of several clones of Ca*gup1Δ *null mutant (3-5) in the presence of some common antifungals and compare them with wt. We used four ergosterol biosynthesis inhibitors (EBIs), hampering different steps of ergosterol biosynthesis [26,27] and two polyenes. The Ca*gup1Δ *null mutant strain was equally sensitive to 25 μg/ml amphotericin B and 2.5 μg/ml nystatin as the wt (see Additional file 1). Conversely, Ca*gup1Δ *null mutant strain displayed a notorious resistance to all the EBIs used, the azoles with antifungal action, clotrimazole, fluconazole and ketoconazole, and the morpholine fenpropimorph (Figure 1). The resistance of Ca*gup1Δ *null mutant strain to clotrimazole and ketoconazole only became obvious at concentrations of 68.8 and 106.3 μg/ml respectively (Figure 1). Moreover, in the presence of 172 μg/ml clotrimazole and of 265.7 μg/ml ketoconazole the growth of both strains was impaired (not shown). The effect of fluconazole, on the other hand, was stronger. The resistance of Ca*gup1Δ *null mutant strain to this drug could be detected using 30.6 μg/ml (Figure 1). With regards to fenpropimorph, we verified that, in the presence of 120 and 240 μg/ml of this drug, none of the strains were able to grow (not shown). When the dosage was reduced to 60 μg/ml, the Ca*gup1Δ *null mutant strain was more resistant than the parental strain (Figure 1). A copy of the *GUP1 *gene, comprising 1.5 Kb of the promoter region and 380 base pairs of the terminator region, was introduced into the Ca*gup1 *null mutant strain at the *RPS1 locus *using the Clp20 plasmid [36]. Correctly, it is possible to see in the same figure that the *GUP1 *complemented strain CF-Ca001, displayed a comparable behaviour to wt. Moreover, the introduction of the empty Clp20 plasmid into Ca*gup1Δ *null mutant, or into wt, did not cause any amendment on these strains phenotypes (not shown).

Furthermore, we checked if the strains had different growth rates, which could have some impact on these results. Indeed, in liquid medium (which is the only way we can compare growth velocities) the doubling time during experimental phase of the wt, mutant and complemented strains is respectively 1.27 ± 0.04 h; 1.43 ± 0.06 h and 1.25 ± 0.05 h. We also determined the mutant doubling time in the presence of fluconazole, which was lower than its value in the absence of the drug. The same happens with the wt. The doubling time during experimental phase of the wt, mutant and complemented strains in the presence of fluconazole are respectively 1.07 1 ± 0.07 h; 1.28 ± 0.09 h and 1.11 ± 0.09 h.

Alternatively, we used the Methyl-Blue diffusion assay. Consistently, wt strain showed halos with wider diameter than Ca*gup1Δ *null mutant strain, indicative that the latter strain is more resistant (see Additional file 2). However, this test provided extra information regarding the nature of inhibition. The halos displayed by the parental strain were dead-halos, in opposition to growth inhibition halos observed with Ca*gup1Δ *null mutant strain (see Additional file 2).

### *CaGUP1 *deletion affects ergosterol distribution

The lower susceptibility of the Ca*gup1Δ *null mutant strain to antifungals prompted us to analyze ergosterol distribution/occurrence in the plasma membrane. The distribution of free cholesterol in mammalian cells can be visualized by fluorescence microscopy using filipin, a fluorescent antifungal compound that interacts with free 3'-β-hydroxy sterols [37,38]. It has been reported, that the use of filipin needs extra cares. It quickly photobleachs, and given its toxicity, it can deform cell membranes upon a prolonged exposure [19,35,39,40]. These problems were overcome using the optimized method, developed by our group before [19].

**Figure 2:**
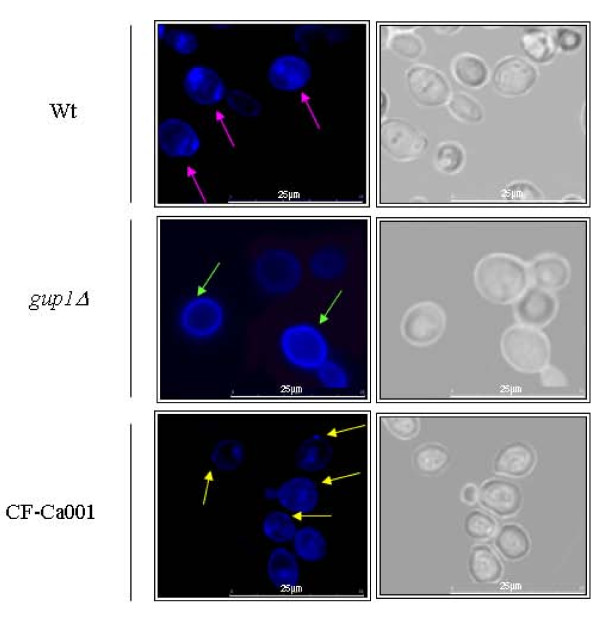
**Sterol lipid distribution is affected by the deletion of Ca*GUP1 *mutation**. The images show filipin staining of the wt, Ca*gup1Δ *null mutant and CF-Ca001 strain cells grown in YPD till mid-exponential phase. Cells were stained with a fresh solution of filipin (5 mg/ml), stabilized onto slides with a drop of an anti-fading agent, and promptly visualized and photographed. Pink and yellow arrows point to punctuated filipin stained sterols at the level of plasma membrane in the wt and CF-Ca001 strains respectively. Green arrows point to filipin stained sterols evenly distributed in the *Cagup1Δ *null mutant plasma membrane. The *gup1Δ *photos are representative of the results obtained with the several clones (3-5) of Ca*gup1Δ *null mutant strain tested.

The pattern of filipin ergosterol staining on the Ca*gup1Δ *null mutant strain differed from the one observed on wt (Figure 2). Overall, fluorescence was mostly present at the cell surface, and Ca*gup1Δ *null mutant strain cells were more intensively stained than wt (Figure 2). As expected [19,39-42], the wt plasma membrane was not stained homogeneously, but rather in distinct patches (Figure 2 - pink arrows). In contrast, filipin-stained sterols distributed homogenously to the Ca*gup1Δ *null mutant strain plasma membrane (Figure 2 - green arrows). The complemented strain, CF-Ca001 displayed a pattern of filipin ergosterol staining similar to wt (Figure 2 - yellow arrows). Conversely, the introduction of the empty Clp20 plasmid into the Ca*gup1Δ *null mutant, or into wt, did not cause any amendment to these strains phenotypes (not shown). These findings indicate that the maintenance and distribution of normal ergosterol levels in the plasma membrane are altered by Ca*GUP1 *deletion.

### Hyphal morphogenesis and colony morphology/differentiation requires *CaGUP1*

**Figure 3:**
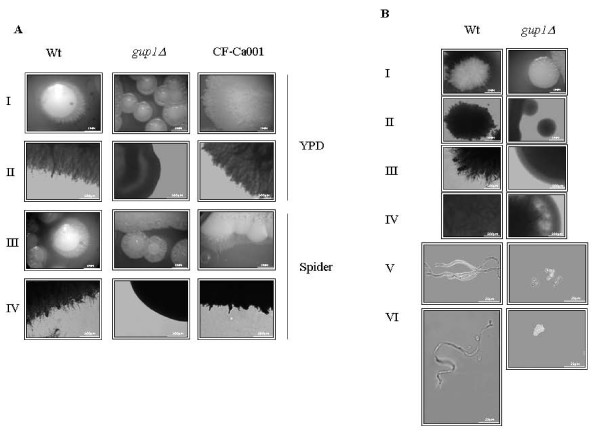
**Ca*gup1Δ *null mutation leads to aberrant colony morphology, precluding filamentous growth**. (A) In both YPD and Spider medium, Ca*gup1Δ *null mutant strain colonies are wrinkled (spaghetti/flower shaped) with no peripheral filamentous growth - panels I and III. The contour of these colonies observed with LM, fully confirms this absence, in clear contrast with wt and CF-Ca001 colonies - panels II and IV. (B) Growth on YPD supplemented with 10% FBS at 37°C yields identical results: colony morphology by magnifying lens (I) and by LM (II), colony contour morphology by LM (III), colony internal structure by LM (IV), and individual cells morphology by LM (V, VI). The *gup1Δ *photos are representative of the results obtained with the several clones (3-5) of Ca*gup1Δ *null mutant strain tested.

In *C. albicans*, the transition between the yeast form and the filamentous forms has long been a very active area of research, mainly due to its involvement in virulence [reviewed by [4,5,7,10,11]]. Furthermore, *C. albicans*, as well as related species, are able to spontaneously and reversibly make the switch between two or more general phenotypes, reflected by distinct colony morphologies [43]. In order to investigate if Ca*GUP1 *was implicated in *C. albicans *morphogenesis, young cultures of wt and Ca*gup1Δ *null mutant strains were cultivated on agar plates under several conditions. Colonies from both strains formed in non-hypha-inducing conditions (YPD at 30°C) are similar in shape, without peripheral hyphae and no hyphal cells within the colony (see Additional file 3). Investigation under hypha-induced conditions presented significant differences between the two strains (Figure 3). In opposition to wt, the colonies of Ca*gup1Δ *null mutant strain did not show filaments, either peripheral or inside the colony, suggesting that the mutant lost the ability to form hyphae under the tested conditions. Furthermore, these colonies show a remarkable distinct/aberrant morphology *i.e*. flower, spaghetti, irregular wrinkled shape when compared to wt. In the same figure it is possible to see that, the *GUP1 *complemented strain CF-Ca001 displayed a comparable behaviour to wt. The introduction of the empty Clp20 plasmid into Ca*gup1Δ *null mutant or into wt did not cause any amendment on these strains morphology (not shown). Most interesting, when visualized under the microscope, cells within the colonies of the mutant strain were all yeast-type (Figure 3B - panel V and VI), and not a mixture of hyphae and blastospores as described in the literature [4,44]. The same pattern was observed irrespectively of the medium used.

**Figure 4:**
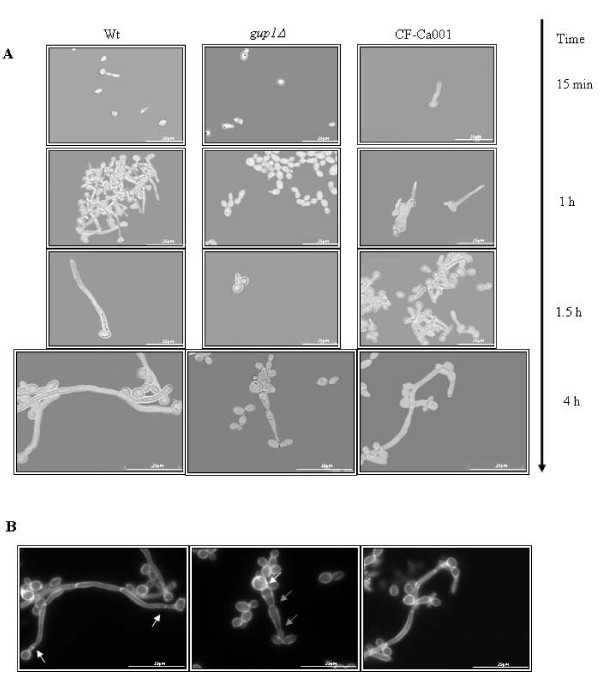
**Ca*gup1*Δ null mutation causes residual filamentous growth in liquid hyphae inducing medium**. (A) Young cell cultures were incubated in liquid YPD with 10% FBS at 37°C. Light microscope samples were photographed at increasing time points. (B) Chitin assembly by CFW staining of the 4 h samples, revealing distinct filament types, hyphae - wt and CF-Ca001 - and pseudohyphae - Ca*gup1*Δ null mutant strain. Arrows indicate the localization of the septa. The *gup1Δ *photos are representative of the results obtained with the several clones (3-5) of Ca*gup1Δ *null mutant strain tested.

Time-course of hyphae formation induced by FBS (fetal bovine serum) in liquid medium was also checked. Wt displayed filamentous growth soon after induction (15 min) (Figure 4A) whereas with the Ca*gup1Δ *null mutant strain this switch was not observed before 1.5 h. During the remaining time of the experiment, filamentous cells from the Ca*gup1Δ *null mutant strain were barely detectable when compared to wt.

Moreover, these filamentous cells were pseudohyphae and not true hyphae as found in wt filamentous cells (Figure 4A, lower panels - time 4 h). Chitin assembly by CFW (Calcofluor white) staining displayed, in the filamentous cells of Ca*gup1Δ *null mutant strain, constrictions at the septae junction (Figure 4B - grey arrows) and at the mother-bud neck, where the first septum is located (Figure 4B - white arrows). In opposition, in the wt filamentous cells, which presented true hyphae, the first septum is distant from the mother neck and the other septa do not present constrictions [reviewed by [4] and by [5]]. Additionally, in contrast to wt, in Ca*gup1Δ *null mutant strain the elongated compartments were thicker, without parallel sides and were highly branched [reviewed by [4] and [5]]. As before, the *GUP1 *complemented strain CF-Ca001, exhibited the same performance as wt (Figure 4), and the control strains with the empty plasmid, act similarly to Ca*gup1Δ *null mutant and wt, correspondingly (not shown). These data support the involvement of *CaGUP1 *in the morphogenic programme required to induce hyphae formation, irrespective of the chosen growth regimen (solid or liquid media).

### Ability of adhesion to polystyrene and invasion of agar is altered on *Cagup1Δ *null mutant

**Figure 5:**
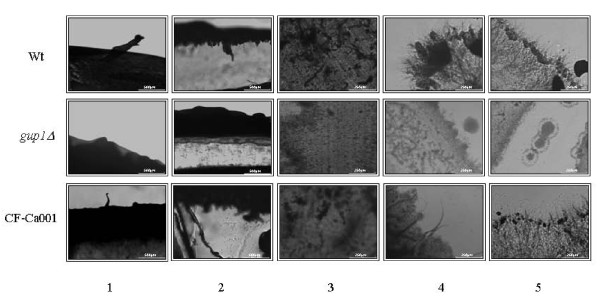
**Ca*gup1*Δ null mutation causes less agar invasiveness/adherence**. Young cultures of *C. albicans *Wt, Ca*gup1Δ *null mutant and CF-Ca001 strains were diluted and spotted onto YPD plates, which were subsequently incubated at 37°C for 5 days. Plates were further washed and the growth remains of washed plates were visualized (1-3). Longitudinal cuts of the grown cultures reveal aerial growth on the agar surface (4) and inwards agar invasion (5). The *gup1Δ *panel photos are representative of the results obtained with the several clones (3-5) of Ca*gup1Δ *null mutant strain tested.

Adhesion of Ca*gup1Δ *null mutant strain cells was tested in two different assays: on agar plates with a plate washing assay [45,46], in both YPD and Spider medium, and on polystyrene through the quantification of total biomass by crystal violet (CV) staining [47-49]. The colonies of Ca*gup1Δ *null mutant strain were found to be washed away much easier from the agar plates than wt or CF-Ca001 colonies (Figure 5- panels 1-3), indicating that the mutant strain cells have a reduced potential to adhere to the agar. Additionally, microscopic observation of agar surface, as well as longitudinal cuts revealing the aerial (Figure 5 - panel 4) and inner (Figure 5 - panel 5) agar/growth limits, shows that the wt and CF-Ca001 hyphae extend to aerial environment, but also penetrate/invade the agar (Figure 5 - panel 4-5). Furthermore, these cells which robustly invaded the agar produced hyphae. On the other hand, the cells of *CagupΔ *null mutant strain were not able to penetrate the agar and failed to form hyphae or pseudohyphae. The introduction of the empty Clp20 plasmid into Ca*gup1Δ *null mutant or into wt did not cause any amendment on these strains phenotypes (not shown). These results suggest that the deletion of *CaGUP1 *abolishes the ability to invade the agar.

**Figure 6:**
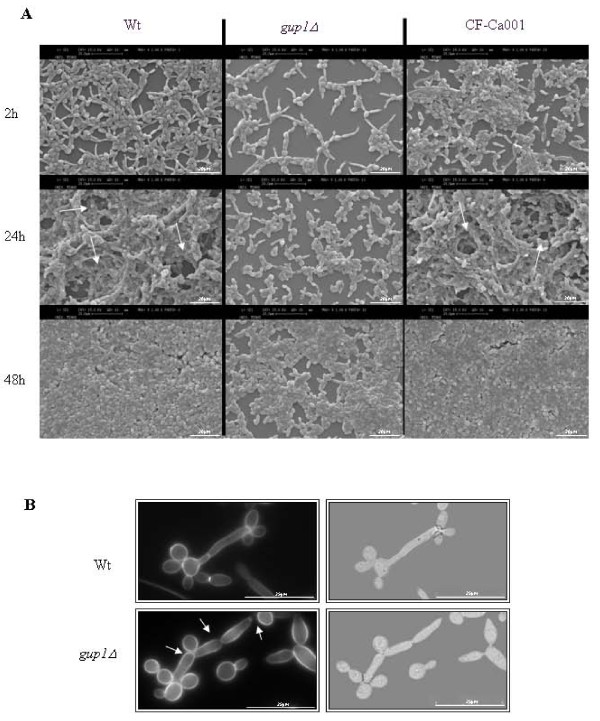
**Ca*gup1*Δ null mutation causes less and differently structured time-course biofilm formation**. (A) SEM micrographs of time course biofilm formation. Arrows indicate the channels observed in a typical biofilm structure - wt and CF-Ca001- not observed in Ca*gup1*Δ null mutant strain biofilm. (B) Chitin assembly by CFW staining of individual cells observed by LM. Distinct filament types can be observed. Wt cells display hyphae without septae constrictions, the first septum located within the germ tube, apart from the mother-bud neck (arrow), and less branched, thinner elongated compartments with parallel sides. Ca*gup1Δ *null mutant strain cells present pseudohyphae with constrictions located at the septae junctions and at the mother-bud neck, where the first septum is located (arrows), highly branched and thicker elongated compartments without parallel sides. The *gup1Δ *photos are representative of the results obtained with the several clones (3-5) of Ca*gup1Δ *null mutant strain tested.

Consonantly, the cells of Ca*gup1Δ *null mutant strain also exhibit lower adherence ability to polystyrene (Table 1), comparing to wt and CF-Ca001 cells. This is evidenced by comparing the absorbance values at 2 h incubation time, reflecting the total adhered biomass, corroborated by SEM observation (Figure 6). Light microscopic observation of these samples revealed an amazing lower number of hyphae/pseudohyphae cells on Ca*gup1Δ *null mutant strain (not shown). The control strains, with empty plasmid, behaved as expected (not shown).

We also inspect the hydrophobicity of the Ca*gup1Δ *null mutant cells, since this factor can influence adhesion. Yet, no significant difference between the % of hydrophobicity of the mutant and wt was observed (2.29% and 2.45% respectively).

### Biofilm formation ability is affected in *Cagup1Δ *null mutant

Both filamentation and adhesion of *C. albicans *are involved in the formation of biofilms [50,51], which are commonly found on medical devices, and have attracted attention because of their persistence and resistance to antifungal agents, contributing to both superficial and systemic candidoses [25,50]. We compared the biofilm forming ability of both wt and Ca*gup1Δ *null mutant strain cells through the quantification of total biomass by crystal violet (CV) staining [47-49] and Scanning Electron Microscopy (SEM). Importantly, Ca*gup1Δ *null mutant strain biofilms had less total biomass compared with wt or with the complemented strain CF-Ca001 (Table 1- absorbance at 24 and 48 h). Wt and the CF-Ca001 strains formed biofilms with biomass ≈ 1.5 times higher than the Ca*gup1Δ *null mutant strain. The biofilm formation ability of the control strain was as expected. Ca*gup1Δ *null mutant strain with the empty Clp20 plasmid, presented the same defect as the mutant and the wt with the empty Clp20 plasmid behaved similarly to wt and the CF-Ca001 (not shown).

**Table 1:** Adhesion and Biofilms Assay

Abs values/cm^2 ^± SD			
**Cell type**	**Time (h)**		
	
	**2**	**24**	**48**

Wt	0.228 ± 0.01	0.324 ± 0.02	0.387 ± 0.06
*gup1*	0.074 ± 0.01	0.222 ± 0.04	0.293 ± 0.02
CF-Ca001	0.209 ± 0.02	0.298 ± 0.02	0.359 ± 0.04

Standardized absorbance values of Crystal Violet solutions (Abs/cm^2^) obtained in adhesion and biofilms assay of Wt, Ca*gup1Δ*, and the control strains (λ = 570 nm).

SEM observation of the same samples reflected these differences (Figure 6). In opposition to wt or the complemented strain CF-Ca001, Ca*gup1Δ *null mutant strain was not able to form typical biofilm structures (Figure 6A). Additionally, Ca*gup1Δ *null mutant strain presented much less hyphae/pseudohyphae cells. On the other hand, cell shape inspection by CFW staining (Figure 6B) showed that the filamentous cells found in wt biofilm were true hyphae, while the filamentous cells of the Ca*gup1Δ *null mutant strain were pseudohyphae (Figure 6B) [4]. As in the induced hyphae experiments (Figure 4), these showed constrictions at the septa and at the mother-bud neck, where the first septum is located, thicker elongated compartments without parallel sides, and highly branched (Figure 6B- white arrows).

## Discussion

In previous works, we showed that *S. cerevisiae *Gup1p, an acyltransferase, is involved in lipids metabolism, with critical consequences on the plasma membrane lipid-ordered domains stability, on the resistance to antifungals [19], as well as in the cell wall constitution, morphology and assembly [32]. These are important features to be considered when regarding both *C. albicans *switch from commensal to pathogen and its increased resistance to antifungal drugs. Our experiments provide compelling evidence that deletion of both *C. albicans GUP1 *alleles promotes resistance to antifungals, similarly to what happens in *S. cerevisiae*, but more importantly, CaGup1p interferes in diverse *C. albicans *virulence factors including hyphal development.

Our assumptions are based on the following observations. First, *Cagup1Δ *null mutant strain is resistant to common antifungals. Second, *CaGUP1 *deletion provokes an aberrant evenly ergosterol distribution at the level of plasma membrane. Third, the ability to switch from yeast-form to hyphal-growth requires *CaGUP1*. Fourth, a distinct growth orientation elicited by the deletion of Ca*GUP1 *leads to colonies with remarkable distinct/aberrant morphology *i.e*. flower, spaghetti, irregular wrinkled shape. Fifth, *Cagup1Δ *null mutant strain adherence and invasion abilities are strongly reduced. Sixth, biofilm formation, another important indicator of *C. albicans *virulence, is strongly impaired by the deletion of *CaGUP1*. Finally, the introduction of the *GUP1 *gene copy into the Ca*gup1Δ *null mutant strain was able to revert all these phenotypes, symptomatic of the *GUP1 *gene accountability.

The *C. albicans *laboratory strain BWP17, has recently been subject of great controversy, due not only to the genomic alterations that occurred in its construction, but also due to URA3 marker [52]. The absence of *URA3 *alleles is associated with several phenotypes, some of them regarding *C. albicans *virulence [36,53]. In this work, we were particularly concerned with this, reason why we considered the use of BWP17 as wt control for *GUP1 *double deletion as more reliable than the mother strain - SC5314. Both BWP17 and *Cagup1Δ *null mutant present the same genetic background, thus overcoming any possible phenotypic side effects derived from altered chromosomal location of the auxotrophic marker. Furthermore, we introduce the *GUP1 *gene copy into the Ca*gup1Δ *null mutant strain using Clp20 plasmid [36], since it additionally expresses *URA3 *and *HIS1 *markers. Integrating vectors are preferable to episomal vectors in *C. albicans*, since they lead to a reduction on the population heterogeneity due to plasmid loss or copy number variance, and this is particularly important for virulence studies. On the other hand, and according to Dennison and co-authors [36], the use of Clp20 plasmid, allows the concomitant regeneration of prototrophy and gene reintegration in null mutants at the *RPS1 *locus. Particularly, the integration of *URA3 *gene at the *RPS1 locus*, circumvent the *URA3 *position effects that can complicate the interpretation of *C. albicans *virulence assays [36,52,53]. Finally, two other control strains Ca*gup1Δ *null mutant and BWP17 with the empty Clp20 plasmid were constructed, and tested, confirming that the introduction of the empty Clp20 plasmid did not cause any amendment on the mutant or on the wt performance, at any level.

It has been shown that subtle modifications on the membrane lipid composition (phospholipids and ergosterol), on its order (fluidity) and asymmetry could be important determinants of yeast cells susceptibility to antifungal drugs [23,24,34]. As already referred, Sc*gup1Δ *mutant presents a distorted lipidic plasma membrane constitution [54], and a changed stability/assembly of the sphingolipids-sterol ordered domains [19]. Furthermore, in Sc*gup1Δ *mutant, ergosterol distribution at the level of plasma membrane is disturbed [19]. As in *S. cerevisiae*, in the Ca*gup1Δ *null mutant strain plasma membrane filipin-stained sterols distributed evenly, in contrast with the usual punctuated distribution found in wt plasma membrane. This may be the basis of the observed increased resistance of Ca*gup1Δ *null mutant strain to the EBIs, fluconazole, ketoconazole and clotrimazole, belonging to the class of azoles, and the morpholine, fenpropimorph. The azoles are antifungals commonly used to treat yeast infections [23,24,27,28,34]. Although in *C. albicans *the lipid biosynthesis pathways are not well documented, in *S. cerevisiae *these drugs operate on the biosynthesis of ergosterol at the C-14 demethylation stage [27,28], causing a combination of ergosterol depletion and the accumulation of lanosterol, along with other 14-methylated sterols [27,28]. Fenpropimorph, as the other morpholines, inhibits two reactions catalyzed by Δ^14 ^reductase (an essential enzyme) and Δ^7^- Δ^8 ^isomerase [27,28], resulting in the accumulation of 24-methylene ignosterol in the plasma membrane [27,28].

Another group of antifungals, the polyenes, in theory interact specifically with the ergosterol present on the plasma membrane [26,55], creating pores and concomitantly provoking plasma membrane physical and functional disruption, and thus cell death. In spite of the changes observed in ergosterol distribution, Ca*gup1Δ *null mutant strain was as sensitive to polyenes as wt. Previous reports, suggest the possibility that polyenes interact also with other membrane lipids besides ergosterol [23,24,34]. In *C. albicans *the metabolism of the other lipids, namely sphingolipids and fatty acids, does not appear to be altered by the deletion of Ca*GUP1*, as can be inferred from the susceptibly of the mutant to these lipids biosynthesis specific inhibitors (Ferreira, C., unpublished results).

In a previous work, we found that the absence of Sc*GUP1 *results in a defective cell wall composition and assembly, with a higher content in β-1,3 glucans and chitin, and lower fraction of mannoproteins [32]. By analogy, and since *C. albicans *and *S. cerevisiae *cell walls are quite alike (with the exception of higher fraction of β-1,6 glucans on the former) [32,56-58], one could considerer the possibility of Ca*gup1Δ *null mutant cell wall also encompasses higher quantities of β-1,3 glucans. In *C. albicans *it was suggested a correlation between cell wall composition/architecture and resistance to azoles, hypha morphogenesis and virulence [59-61]. Namely, a putative role in azoles resistance on biofilm cells has been ascribed to β-1,3- glucans [61]. Nett and co-authors described cell wall architectural changes, and increased β-1,3 glucans content associated with fluconazole resistance [61].

Cell wall dynamics in *C. albicans*, underlie regulatory processes during the yeast-to-hyphae transition [59-63]. The ability to switch rapidly between these two forms of growth is a defining characteristic of *C. albicans *cells. Nevertheless, each form of growth provides critical functions required for pathogenicity/virulence [reviewed by [4] and by [5,7]]. Namely, hyphae form is thought to facilitate host tissues invasion and escape from phagocytotic destruction [reviewed by [4] and by [5,7,64]]. We found that, Ca*gup1Δ *null mutant strain lost completely the ability to differentiate into hyphae when cultured in solid inducing media conditions, and suffered a considerable delay to grow as filaments when induced with serum in liquid cultures. In this last case, the few remaining Ca*gup1Δ *null mutant filamentous cells were smaller, and showed to be pseudohyphae and not true hyphae. When a copy of the *GUP1 *gene was introduced into Ca*gup1Δ *null mutant, the resulting strain CF-Ca001 regained the ability to differentiate into hyphae, as wt reflecting the role of *GUP1 *gene. Interestingly, mammalian *GUP1 *gene [33] was able to complement hyphal development defects of Ca*gup1Δ *null mutant (Ferreira, C., unpublished results).

The aberrant shape of the Ca*gup1Δ *null mutant strain colonies (flower, spaghetti, irregular wrinkled shape) did not present any filamentous cells. This is in accordance with the observed Ca*gup1Δ *null mutant defect to grow into hyphae, but appears to be in disagreement with the literature, that attributes a mixture of yeast and hyphae cells to these colonies [reviewed by [4,65,66]]. The complex morphology of these colonies depends, besides other factors, on polarized growth orientation [reviewed by [5,62,63]], which was found to be altered in *Scgup1Δ *mutant [30,32]. Additionally, we cannot disregard the possibility that these morphologic cues, may derive from the contribution of the miss-localization/impaired function of specific plasma membrane/wall sensor/proteins.

Invasiveness depends on the existence of hyphae and/or pseudohyphae cells [4]. Accordingly, wt and CF-Ca001 cells were able to invade the agar, whereas Ca*gup1Δ *null mutant strain cells lost this ability. This is of extreme relevance in tissue penetration during the early stages of infection. The yeast form might be more suited for dissemination in the bloodstream [4]. Other crucial features with a clear impact on *C. albicans *pathogenicity are the adherence and biofilm formation abilities. The adhesion of Ca*gup1Δ *null mutant strain cells either to agar or to polystyrene was greatly reduced when compared to wt and CF-Ca001 strains, which in the former case is in accordance with a lesser agar invasion, due in part to the lack of filamentous growth. The hydrophobicity of the cells can also influence adhesion, yet Ca*gup1Δ *null mutant strain hydrophobicity does not differ from wt. So, their dissimilarities in terms of adherence cannot be explained by this property. However, it is important to highlight that the adhesion phenomenon is not only dependent of cell wall hydrophobicity. Other factors may contribute significantly to it, such as the cell wall charge, cell wall composition (in terms of proteins or other components) [reviewed by [67]] and even the yeast morphology. Moreover, there are many reports acknowledging the inconsistency between the adherence ability and strain hydrophobicity, particularly in *C. albicans *and non-*albicans *isolated strains but also, in other microorganisms as is the case of bacteria [49,68-71].

According to some authors [51,72] three basic stages are necessary for *Candida *biofilm formation *in vitro*: 1) attachment of yeast cells to the surface, 2) growth and proliferation of yeast cells to form a basal layer, followed by 3) extensive filamentation combined with the production of extracellular matrix. We observed a strong defect on the ability of Ca*gup1Δ *null mutant strain to form biofilm on an inert substrate (polystyrene wells). The attachment of Ca*gup1Δ *null mutant strain cells to this surface, *i.e*. their adherence was nearly one third than the parent strain and no differentiated structure was formed. These observations corroborate defects in the 2 first basic stages above mentioned. Additionally, also the 3^rd^, *i.e*. extensive filamenttation was highly compromised.

## Conclusions

In conclusion, we demonstrate that in Ca*gup1Δ *null mutant strain the major virulence factors are severely weakened, namely the impaired ability of form true hyphae, to adhere and to invade to different substrates and form biofilms. Equally important, was the revealing role of Ca*GUP1 *gene in the resistance to antifungals. The present work brings cutting-edge insights into the role of Gup1p on the transformation of *C. albicans *into a pathogen. All taken, and considering the fact that mm*GUP1 *gene complemented the hyphal morphogenetic defects of Ca*gup1Δ *null mutant (Ferreira, C., unpublished results); we anticipate that Gup1p may be part of a yeast morphogenic pathway parallel to the mammalian Hedgehog.

## Methods

### Yeast strains, media and growth conditions

*C. albicans *strains used in this work were BWP17 (*ura3*Δ*::λimm434/ura3*Δ *::λimm434his1::hisG/his1::hisGarg4::hisG/arg4::hisG*) [73], several clones (3-5) of homozygous *C. albicans gup1Δ/gup1Δ *(isogenic to BWP17 but *gup1::URA3-dpl200/gup1::ARG4*) [74], and CF-Ca001 (isogenic to *C. albicans gup1Δ/gup1Δ::GUP1*) (this study). All assays were preceded by batch cultures grown on complex medium (YPD: 1% (w/v) yeast extract; 2% (w/v) peptone), supplemented with 2% (w/v) glucose as carbon and energy source, at 26°C to maintain unicellular yeast form. These cultures were continuously inspected as to the absence of hyphae - referred ahead as young cultures. Incubation was done at 160 rpm, orbital shaking with air/liquid ratio 2.5/1. Growth was monitored spectrophotometrically at 600 nm. Solid media were supplemented with 2% (w/v) agar.

Induction of hyphal growth was as follows: Young YPD cultures (above) were inoculated into YPD, YPD + 10% FBS or Spider's medium [1% (w/v) nutrient broth, 1% (w/v) mannitol, 0.2% (w/v) K_2_HPO_4 _[75]], supplemented with 1.5% agar, and grown at 37°C for 3-5 days. For time-course induction with FBS in liquid broth, cells from young cultures were washed, resuspended (1 × 10^7 ^cell/ml) in YPD supplemented with 10% FBS and incubated at 37°C. Photomicrographs were taken at representative time-points.

### Strain construction

To reintroduce *GUP1 *into *C. albicans gup1Δ/gup1Δ*, the *GUP1 *locus was PCR amplified with QIAGEN LongRange PCR Kit (Qiagen) using the primers ACGCGTCGCTACTTCACATGGTATAAGTGTTGCTGATTTGG and GATTAATA TCAAATTTTTCAACCAAGCAAGCATTCAGCTG (MluI and SalI sites underlined), and cloned into the plasmid CIp20 [36] using CloneEZ^® ^PCR Cloning Kit (Genscript). CIp20, which is a derivative of CIp10 [76], contains the *URA3 *and *HIS1 *markers. CIp20-*GUP1 *was linearized with StuI, transformed into *C. albicans gup1Δ/gup1Δ *to create the *GUP1*-reintegrant strain CF-Ca001. The integration of CIp20-*GUP1 *at the *RPS1 locus *was confirmed by PCR with primers TTGTATCACAACCCTCCC and GTGGTTGGAGCTTTGATG. The control strains were generated by transforming the parental strain (BWP17) and the homozygous *C. albicans gup1Δ/gup1Δ *with the empty CIp20 plasmid linearized with StuI.

### Sensitivity to lipid biosynthesis inhibitors

#### (i) Drop tests

Drop tests were performed from YPD cellular young cultures suspensions, containing approximately 1 × 10^6 ^cells/ml. Ten-fold serial dilutions were made, and 5 μl of each suspension was applied on the selective media. Results were scored after 3-5 days of incubation at 30°C. Selective conditions were as follow: clotrimazole (68.8 and 172 μg/ml), ketoconazole (106.3 and 265.7 μg/ml), fluconazole (30.6, 91.8 and 153 μg/ml) and fenpropimorph (60, 120 and 240 μg/ml), amphotericin B (25 μg/ml) and nystatin (2.5 μg/ml). All chemicals were obtained at the highest available grade from Sigma Aldrich.

#### (ii) Methyl-blue diffusion test

Alternatively, we assayed the sensitivity to lipid biosynthesis inhibitors with a methyl-blue-diffusion plate test. Sterile filter disks (BBL) of 6 mm diameter were placed on top of YPD methyl-blue plates seeded with 5 ml of a wt or Ca*gup1Δ *mutant strain young cultures. The filter disks were impregnated with 5 to 10 μl of the following drugs: clotrimazole (137.6 μg/ml), ketoconazole (212.6 μg/ml), fluconazole (91.8 μg/ml), fenpropimorph (80 μg/ml), amphotericin B (25 μg/ml) and nystatin (2.5 μg/ml). The plates were incubated at 30°C, and halos of inhibition were scored after 3 days. Again, all chemicals were obtained at the highest available grade (Sigma-Aldrich).

### Filipin/Sterol fluorescence microscopy

Sterol-lipid distribution was assessed *in vivo *using filipin. This was performed basically as described before [19,40]. For fluorescence microscopy, cells were mounted directly on slides with a 10 μl drop of anti-fading agent Vectashield (Vector Laboratories) to overcome the instability of filipin, and immediately observed by light microscopy (LM).

### Colony morphology and differentiation

To observe different colony morphology/differentiation, equal volumes of young cultures of each strain were diluted and spotted onto non-inducing (YPD at 30°C) and hyphal-inducing (Spider medium and on 10% FBS at 37°C) conditions, and also in YPD at 37°C. Cultures were allowed to grow for 3-5 days. Colonies on agar surface were observed under magnifying lens (10 times) and photographed. Spider medium colonies were also thoroughly observed by light microscopy. To visualize cellular morphology within the colony, cells of each colony phenotype were resuspended and washed twice in PBS. Approximately 10 μl of the suspensions were then mounted on glass slides and cells were visualized by LM.

### Chitin assembly analysis

To discriminate between hyphae and pseudohyphae cell wall chitin assembly was assessed with CFW staining. Cultures were diluted to 1 × 10^7 ^cells/ml and to 1 ml of cells suspension was added 100 μl of CFW (300 μg/ml). Samples were incubated at room temperature for 5 min and 5 μl of each suspension placed on glass slide for microscopic inspection. The dye fluoresces when bound to chitin, primarily, and to glucans, staining cell wall and septa. Representative images were obtained by LM.

### Adherence to agar and invasion capacities

Equal volumes of young cultures of each strain were diluted to 1 × 10^7 ^cells/ml, and 1 ml of cells suspension was spotted onto YPD medium agar plates. Solid cultures were allowed to grow at 37°C for 5 days. The cells on the surface were removed by washing under running water [45,46] and then visualized by LM. Inspection of agar invasion was performed by visualization of longitudinal cuts displaying the aerial and internal agar/growth boundaries by LM.

### Light microscopy

Microscopy assessments were done in a Leica Microsystems DM-5000B epifluorescence microscope, with appropriate filter settings. Images were acquired through a Leica DCF350FX digital camera and processed with LAS AF Leica Microsystems software.

### Cell wall hydrophobicity

MATH test was utilized to evaluate cell wall hydrophobicity as described by Rosenberg [77]. Yeast cells were harvested in stationary phase and washed twice with PBS pH 7.0. A yeast cell suspension displaying an optical density at 600 nm (OD_600 nm_) between 0.4-0.5 was prepared in PBS (A_0_). In an acid washed spectrophotometer glass tubes, 3 ml of the prepared yeast suspension was spread and overlaid by 0.4 ml of a hydrophobic hydrocarbon, hexadecane. After vigorous vortexing, phases were allowed to separate for 10 min at 30°C and OD_600 nm _of the aqueous phase was measured (A_1_). The percentage of hydrophobicity was calculated as follows: hydrophobicity (%) = [1-(A_1_/A_0_)] × 100. Assays were performed in triplicate and statistical analysis (T-test, p < 0.05) of the results was carried out.

### Adhesion and biofilm formation

Adhesion and biofilm formation ability was assessed through quantification of total biomass by crystal violet (CV) staining as described before [47-49]. For this, standardized cell suspensions (1 ml containing 1 × 10^7 ^cells/ml in YPD) from young cultures were placed into selected wells on polystyrene plates (Orange Scientific, Braine-l'Alleud, Belgium) and incubated at 37°C in a shaker at 120 rev/min. Adhesion ability was measured after 2 h of incubation and biofilm formation ability was inspected after 24 h and 48 h. Regarding the 48 h sample, an extra step was performed, at half period, *i.e*. at 24 h, 500 μl of YPD medium were removed and an equal volume of fresh YPD added. After the defined times of incubation, the medium was aspirated and non-adherent cells removed by washing the wells with sterile ultra-pure water. Following, the adherent cells were fixed with 1 ml of methanol, which was removed after 15 min of contact. The plates were allowed to dry at room temperature, and 1 ml of CV (1% v/v) was added to each well and incubated for 5 min. The wells were then gently washed with sterile, ultra-pure water, and 1 ml of acetic acid (33% v/v) was added to release and dissolve the stain. The absorbance of the obtained solution was read at 570 nm in triplicate in a microtiter plate reader (Bio-Tek Synergy HT, Izasa, Lisbon, Portugal). The final absorbance was standardized according to the volume of acetic acid and area of the wells (abs/cm^2^). Three to five independent assays were performed for each experiment.

### Scanning electron microscopy

Structure of adhered and/or biofilm cells were examined by Scanning Electron Microscopy (SEM). For this, medium and non-adherent cells were extracted as described for CV staining (above). Samples were then dehydrated with alcohol (using 70% ethanol for 10 min; 95% ethanol for 10 min and 100% of ethanol for 20 min) and air dried for 20 min. The bases of the wells were cut and were kept in a desiccator until analysed. Samples were then covered with gold for visualization in a S-360 scanning electron microscope (Leo, Cambridge, USA).

## Authors' contributions

CF conceived the project, performed EBI's sensibility, filipin, hyphal development, morphology, microscopy (light and SEM), adhesion and invasion abilities experiments and wrote the paper; SS and MH conceived and performed biofilm experiments; SS also participate in SEM experiments; EP and FFO performed complementation experiments; CL provided the founds and executed critical readings of the manuscript at several stages. All authors have read and approved the final manuscript.

## Supplementary Material

Additional file 1 **Growth inhibition halos in the presence of polyenes**. Sterile filter disks were impregnated with 25 μg/ml amphotericin B (AmpB) and 2.5 μg/ml nystatin (Nys) and placed on top of YPD methyl-blue plates seeded with 5 ml of a wt or Ca*gup1Δ *null mutant strain mid-exponential phase cultures. Halos of growth inhibition were measured (mm) after 2 or 3 days. Click here for file

Additional file 2 **Growth inhibition halos in the presence of EBIs**. Sterile filter disks were impregnated with the drugs and placed on top of YPD methyl-blue plates seeded with 5 ml of a wt or Ca*gup1Δ *null mutant strain mid-exponential phase cultures. (1) YPD plates (control) and plates with the impregnated disks (2) clotrimazole 137.6 μg/ml, (3) ketoconazole 212.6 μg/ml, (4) fluconazole 91.8 μg/ml and (5) fenpropimorph 80 μg/ml. Halos of growth inhibition were measured (mm) after 2 or 3 days. Click here for file

Additional file 3 **Colony morphology under non-hypha-inducing conditions**. Ca*gup1Δ *null mutant and wt present similar colony morphology in non-hypha-inducing conditions. Equal volumes of young cultures of each strain were diluted and spotted onto YPD, and allowed to grow at 30°C for 3-5 days. Click here for file
