# Supplementary figures and images for: Candida albicans virulence and drug-resistance requires the O-acyltransferase Gup1p
Source: BMC Microbiol. 2010 Sep 15;10:238. doi: 10.1186/1471-2180-10-238 (PMC2945937; doi:10.1186/1471-2180-10-238)

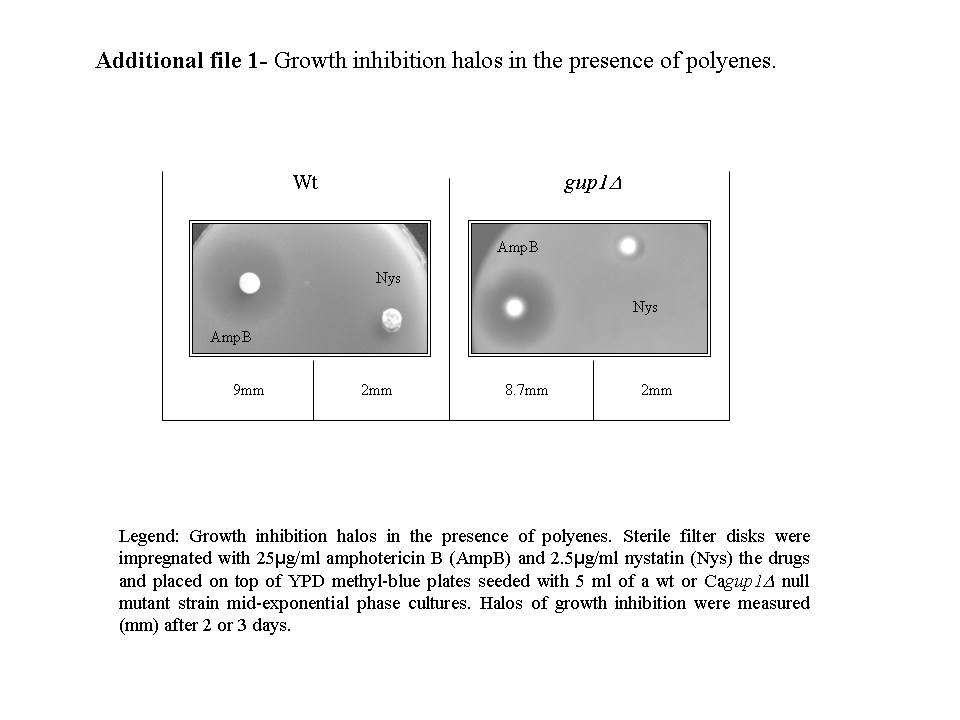

Supplement: Additional file 1 — Growth inhibition halos in the presence of polyenes. Sterile filter disks were impregnated with 25 μg/ml amphotericin B (AmpB) and 2.5 μg/ml nystatin (Nys) and placed on top of YPD methyl-blue plates seeded with 5 ml of a wt or Cagup1Δ null mutant strain mid-exponential phase cultures. Halos of growth inhibition were measured (mm) after 2 or 3 days. [file 1471-2180-10-238-S1.PNG]

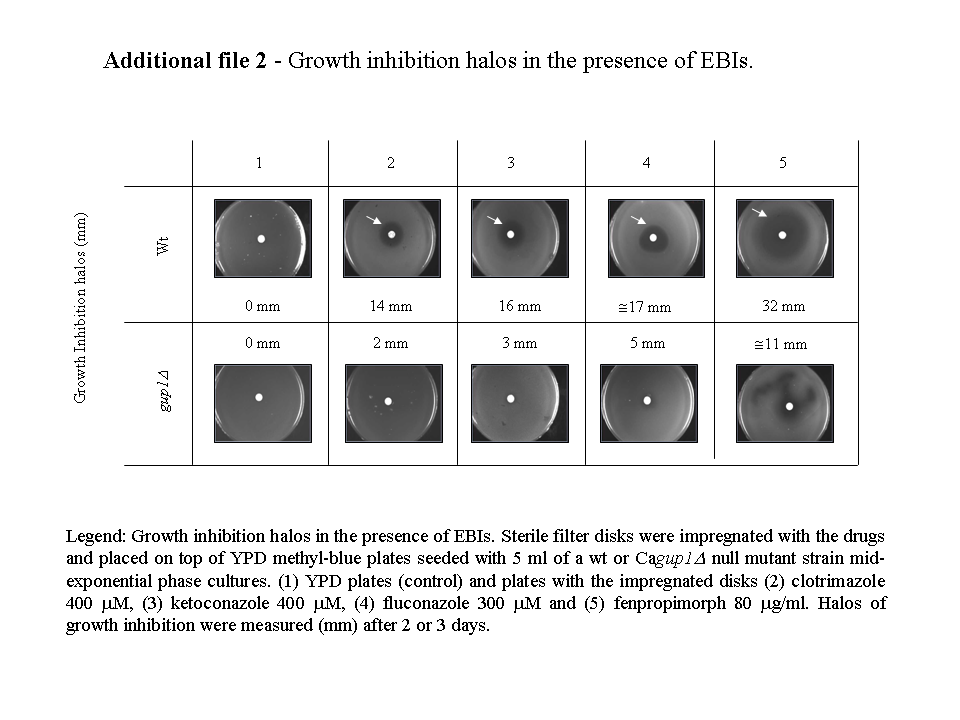

Supplement: Additional file 2 — Growth inhibition halos in the presence of EBIs. Sterile filter disks were impregnated with the drugs and placed on top of YPD methyl-blue plates seeded with 5 ml of a wt or Cagup1Δ null mutant strain mid-exponential phase cultures. (1) YPD plates (control) and plates with the impregnated disks (2) clotrimazole 137.6 μg/ml, (3) ketoconazole 212.6 μg/ml, (4) fluconazole 91.8 μg/ml and (5) fenpropimorph 80 μg/ml. Halos of growth inhibition were measured (mm) after 2 or 3 days. [file 1471-2180-10-238-S2.PNG]

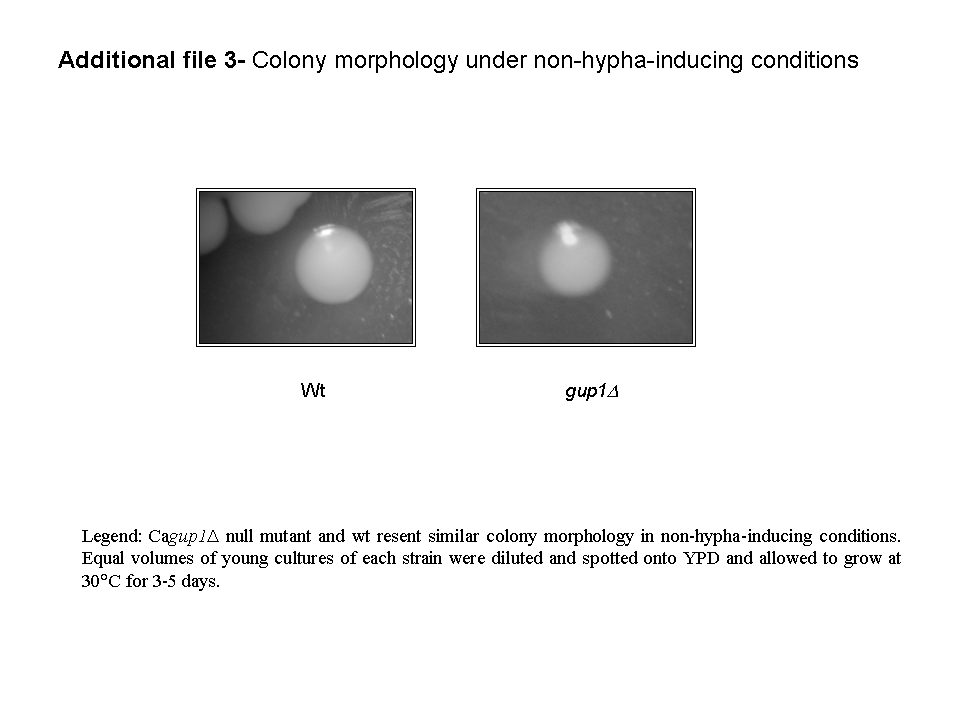

Supplement: Additional file 3 — Colony morphology under non-hypha-inducing conditions. Cagup1Δ null mutant and wt present similar colony morphology in non-hypha-inducing conditions. Equal volumes of young cultures of each strain were diluted and spotted onto YPD, and allowed to grow at 30°C for 3-5 days. [file 1471-2180-10-238-S3.PNG]
